# Supplementary material for: An open-source framework for large-scale, flexible evaluation of biomedical text mining systems
Source: J Biomed Discov Collab. 2008 Jan 29;3:1. doi: 10.1186/1747-5333-3-1 (PMC2276192; doi:10.1186/1747-5333-3-1)
Supplement: Additional file 1 — Evaluation platform source code. The additional file contains the software evaluation framework discussed in this paper. The most current release can be downloaded from SourceForge.net [1]. See the accompanying README file for instructions on installation and use. Also included in the distribution is a collection of UIMA wrappers for some commonly used BioNLP tools and annotated corpora. [file 1747-5333-3-1-S1.zip › bionlp_uima_repository-1.0.1/docs/api/src-html/edu/uchsc/ccp/util/nlp/annotation/comparison/AnnotationComparator.html]

```
001    package edu.uchsc.ccp.util.nlp.annotation.comparison;
002    
003    import java.util.ArrayList;
004    import java.util.Comparator;
005    import java.util.List;
006    
007    import edu.uchsc.ccp.util.nlp.annotation.TextAnnotation;
008    import edu.uchsc.ccp.util.nlp.mention.comparison.ClassMentionComparator;
009    import edu.uchsc.ccp.util.nlp.mention.comparison.IdenticalClassMentionComparator;
010    import edu.uchsc.ccp.util.nlp.stats.PRFResult;
011    
012    public class AnnotationComparator implements Comparator<TextAnnotation> {
013    
014        /**
015         * Returns negative integer, zero, or a positive integer. Zero indicates that the two TextAnnotation objects are equal given the comparison
016         * criteria. The negative and positive integer serve as an aggregate score derived from comparing both the span and class of each TextAnnotation.<br>
017         * <br>
018         * Span comparison results in 0 if the spans are equal given the comparison criteria, -1 of the span(s) from the first TextAnnotation preceeds the
019         * span(s) of the second TextAnnotation, and 1 otherwise.<br>
020         * <br>
021         * The class comparison returns larger number. 0 is returned for classes that match given the comparison criteria, however, -3 is returned
022         * TextAnnotation according to their spans.<br>
023         * <br>
024         * The AnnotationComparator returns an aggreate of the span and class comparison operations, thus enabling the ultimate result to be deciphered
025         * externally of the Annotation Comparator. The default comparison utilizes the StrictSpanComparator and the IdenticalClassComparator
026         */
027    
028        /**
029         * Default TextAnnotation comparison utilizes the StrictSpanComparator and the IdenticalMentionComparator family
030         */
031        public int compare(TextAnnotation ta1, TextAnnotation ta2) {
032            return compare(ta1, ta2, new StrictSpanComparator(), new IdenticalClassMentionComparator());
033        }
034    
035        /**
036         * Compares two TextAnnotations using the supplied SpanComparator and ClassMentionComparator
037         * 
038         * @param ta1
039         * @param ta2
040         * @param spanComparator
041         * @param classMentionComparator
042         * @return
043         */
044        public int compare(TextAnnotation ta1, TextAnnotation ta2, SpanComparator spanComparator, ClassMentionComparator classMentionComparator) {
045            return compare(ta1, ta2, spanComparator, classMentionComparator, -1);
046        }
047    
048        /**
049         * Default comparison of two lists of TextAnnotations utilizing the StrictSpanComparator and the IdenticalMentionComparator family
050         * 
051         * @param taList1
052         * @param taList2
053         * @return
054         */
055        public PRFResult compare(List<TextAnnotation> goldTaList, List<TextAnnotation> taList) {
056            return compare(goldTaList, taList, new StrictSpanComparator(), new IdenticalClassMentionComparator());
057        }
058    
059        /**
060         * Compares two lists of TextAnnotations using the supplied SpanComparator and ClassMentionComparator
061         * 
062         * @param taList1
063         * @param taList2
064         * @param spanComparator
065         * @param classMentionComparator
066         * @return
067         */
068        public PRFResult compare(List<TextAnnotation> goldTaList, List<TextAnnotation> taList, SpanComparator spanComparator,
069                ClassMentionComparator classMentionComparator) {
070            return compare(goldTaList, taList, spanComparator, classMentionComparator, -1);
071        }
072    
073        /**
074         * Compares two TextAnnotations using the supplied SpanComparator and ClassMentionComparator, but only compares to the supplied comparison depth.
075         * 
076         * @param ta1
077         * @param ta2
078         * @param spanComparator
079         * @param mentionComparator
080         * @return
081         */
082        public int compare(TextAnnotation ta1, TextAnnotation ta2, SpanComparator spanComparator,
083                ClassMentionComparator classMentionComparator, int maximumComparisonDepth) {
084            /* span comparison is done during class comparison by default, therefore this is somewhat redundant.. */
085            int spanComparisonResult = spanComparator.compare(ta1.getSpans(), ta2.getSpans());
086    
087            int mentionComparisonResult = classMentionComparator.compare(ta1.getClassMention(), ta2.getClassMention(), spanComparator,
088                    maximumComparisonDepth);
089    
090            int metaDataComparisonResult = metaDataCompare(ta1, ta2);
091    
092            return spanComparisonResult + mentionComparisonResult + metaDataComparisonResult;
093        }
094    
095        /**
096         * Compares two lists of TextAnnotations using the supplied SpanComparator and ClassMentionComparator, but only compares to the supplied
097         * comparison depth.
098         * 
099         * @param taList1
100         * @param taList2
101         * @param spanComparator
102         * @param classMentionComparator
103         * @param maximumComparisonDepth
104         * @return
105         */
106        public PRFResult compare(List<TextAnnotation> goldTaList, List<TextAnnotation> testTaList, SpanComparator spanComparator,
107                ClassMentionComparator classMentionComparator, int maximumComparisonDepth) {
108    
109            boolean[] foundMatchForGold = new boolean[goldTaList.size()];
110            boolean[] foundMatchForTest = new boolean[testTaList.size()];
111            int fn = 0;
112    
113            /* keep track of the tp, fp, and fn annotations */
114            List<TextAnnotation> tpAnnotations = new ArrayList<TextAnnotation>();
115            List<TextAnnotation> fpAnnotations = new ArrayList<TextAnnotation>();
116            List<TextAnnotation> fnAnnotations = new ArrayList<TextAnnotation>();
117            
118            
119            for (int i = 0; i < goldTaList.size(); i++) {
120                TextAnnotation goldTA = goldTaList.get(i);
121                for (int j = 0; j < testTaList.size(); j++) {
122                    TextAnnotation testTA = testTaList.get(j);
123    
124                    /* compare the two TextAnnotations */
125                    int comparisonResult = compare(goldTA, testTA, spanComparator, classMentionComparator, maximumComparisonDepth);
126    
127                    if (comparisonResult == 0) {
128                        /* we have found an exact match, so mark that appropriate gold annotation as matched */
129                        foundMatchForGold[i] = true;
130                        foundMatchForTest[j] = true;
131                    }
132                }
133    
134                /*
135                 * after cycling through all of the annotations in the testTAList, if we did not find a match for this particular goldTA, then we need to
136                 * increment the False Negative count
137                 */
138                if (!foundMatchForGold[i]) {
139                    fn++;
140                    fnAnnotations.add(goldTA);
141                }
142            }
143    
144            /*
145             * now that we have cycled through the entire set of annotations, we can compute the number of True Positives and False Positives by examining
146             * the foundMatchForTest array
147             */
148            int tp = 0;
149            int fp = 0;
150            for (int i = 0; i < foundMatchForTest.length; i++) {
151                if (foundMatchForTest[i]) {
152                    tp++;
153                    tpAnnotations.add(testTaList.get(i));
154                } else {
155                    fp++;
156                    fpAnnotations.add(testTaList.get(i));
157                }
158            }
159    
160            
161            PRFResult prfResult = new PRFResult(tp, fp, fn);
162            prfResult.setTPAnnotations(tpAnnotations);
163            prfResult.setFPAnnotations(fpAnnotations);
164            prfResult.setFNAnnotations(fnAnnotations);
165            
166            return prfResult;
167        }
168    
169        /**
170         * Compare the meta data of two annotations (document ID, document collection ID, and span(s)). A warning is issued if it is determined that two
171         * annotations do not have identical meta data
172         * 
173         * @param ta
174         * @return
175         */
176        public int metaDataCompare(TextAnnotation ta1, TextAnnotation ta2) {
177            /* document IDs must be equal */
178            boolean equalDocumentID = ta1.getDocumentID().equals(ta2.getDocumentID());
179    
180            /* document collection ids must be equal */
181            boolean equalDocumentCollectionID = (ta1.getDocumentCollectionID() == ta2.getDocumentCollectionID());
182    
183            if (equalDocumentID & equalDocumentCollectionID) {
184                return 0;
185            } else {
186    //            warn("Detected the attempted comparison of two annotations with different meta data, implying they are from different document collections, or different documents.");
187                return -1000;
188            }
189        }
190    
191    //    
192    
193    
194    }
```
